# Current Status of Needles in the Optimization of Endoscopic Ultrasound-Guided Procedures

**DOI:** 10.3390/diagnostics10070463

**Published:** 2020-07-08

**Authors:** Akashi Fujita, Shomei Ryozawa, Yuki Tanisaka, Tomoya Ogawa, Masahiro Suzuki, Tatsuya Noguchi, Hiromune Katsuda, Masafumi Mizuide

**Affiliations:** Department of Gastroenterology, Saitama Medical University International Medical Center, 1397-1, Yamane, Hidaka, Saitama 350-1298, Japan; a.fujita0628@gmail.com (A.F.); tanisaka1205@gmail.com (Y.T.); t.ogawa0210@icloud.com (T.O.); msuzgast@tmd.ac.jp (M.S.); k055eb@yahoo.co.jp (T.N.); goobygoobygoobygoo@gmail.com (H.K.); mizuide1971@yahoo.co.jp (M.M.)

**Keywords:** endoscopic ultrasound, endoscopic ultrasound-guided fine-needle aspiration, fine needle biopsy, diagnostic accuracy, interventional endoscopic ultrasound, needle-based confocal laser endomicroscopy, through-the-needle forceps biopsy

## Abstract

Endoscopic ultrasound (EUS) is among the most important tools for the evaluation of gastrointestinal tumors and affected areas around the gastrointestinal tract. It enables the acquisition of material from abnormal lesions via the gastrointestinal wall for tissue confirmation via endoscopic ultrasound-guided fine-needle aspiration (EUS-FNA). EUS-FNA has played a vital role in oncological care and has become the standard method for tissue sampling. The choice of needle type is an important factor determining tissue acquisition and has been evaluated by many researchers. New needles are introduced into the market almost every year, and opinions vary regarding proper needle selection. While there are diverse opinions but no definitive recommendations about the use of one particular device, fine-needle biopsy needles may provide detailed information on a tissue’s architecture based on greater sample yields. This permits additional analyses, including genetic sequencing and phenotyping, thereby enabling the provision of more personalized treatment plans. Furthermore, other EUS-guided procedures have been developed, including interventional EUS and through-the-needle devices. Given the continued attempts to improve the diagnostic ability and therapeutic techniques, we review in detail the available types of puncture needles to provide guidance on the selection of the appropriate needle types.

## 1. Introduction

Endoscopic ultrasound (EUS) is one of the important tools for evaluation of gastrointestinal tumors and affected areas around the gastrointestinal tract. It has great advantages over CT and other imaging modalities because it enables assessment of the echo structure in < 1 cm diameter lesions. In addition, endoscopic ultrasound-guided fine-needle aspiration (EUS-FNA) provides the capability to obtain material from abnormal lesions via the gastrointestinal wall for tissue analysis [1].

**Figure 1:**
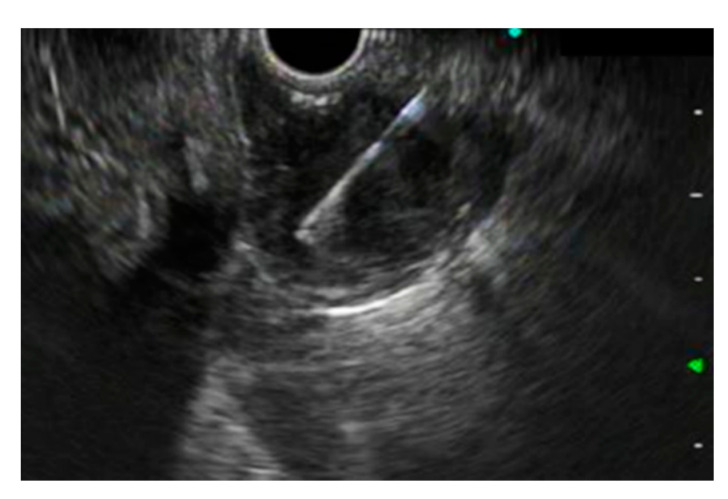
Endoscopic ultrasound-guided fine-needle aspiration (EUS-FNA) of a gastric subepithelial lesion.

This safe and widely used tissue sampling procedure provides consistent results under ultrasound guidance [2,3,4], and it is thought to be more effective compared to CT-guided or ultrasound-guided biopsy of lesions [5]. EUS-FNA has been mainly used for pancreatic solid lesions [6,7], abdominal or mediastinal lymph nodes [8,9,10], and gastrointestinal subepithelial lesions (SELs) (Figure 1) [3,11].

More recently, its range of indications has expanded so as to include liver lesions [12], adrenal grand lesions [13], and biliary strictures [14,15]. For pancreatic cystic lesions, indications vary significantly between countries due to the risk of dissemination [16,17]. EUS-FNA enables the acquisition of histological evidence of cancer when chemotherapy is being considered, to distinguish benignancy or malignancy when deciding if surgery or follow-up is needed, and assessment of the degree of progression of malignant tumors when unexplained lymph-node swelling is detected. The sensitivity and specificity of EUS-FNA for detecting solid pancreatic lesions have been estimated to lie between 85–89% and 96–99%, respectively [18,19,20]. Although there may be complications such as pancreatitis and bleeding, EUS-FNA has been very safe, with < 1% of morbidity and mortality rates [21].

**Figure 2:**
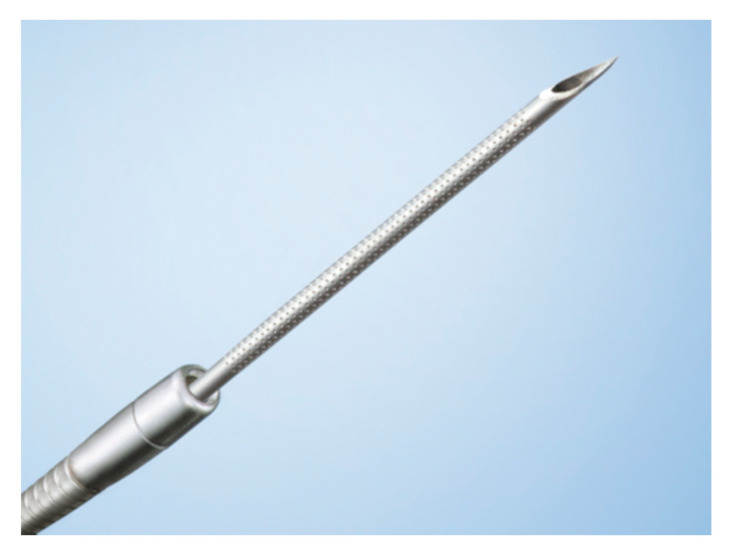
The Ez shot3 (Olympus Medical Systems, Tokyo, Japan) with a Menghini shape of the needle tip (Image courtesy of Olympus Medical Systems).

Several factors affect the outcome of this technique, such as the exact location and characteristics of the lesion, the skill level of the endoscopist, the used sampling technique, availability of rapid on-site evaluation (ROSE), and needle gauge or type [22]. Selection of the needle is considered to be an essential factor, which affects the quality of tissue acquisition and has consequently been evaluated in several studies. A diversity of needles has been in use over the past 25 years, supported by a continuous stream of design innovations. New needle designs are introduced almost annually, and views on the proper needle selection are wide-ranging (Figure 2), partly because they are used not only for tissue sampling but also for various interventional procedures.

This review focuses on past and current advances in design and status of needles and other devices that are used in EUS-guided procedures.

## 2. The History of Needle Development 

EUS has made great progress over the past decades. In the late 1970s and 1980s, it was developed by the Olympus Corporation as an improvement to ultrasound imaging techniques of the pancreaticobiliary system. The initial EUS prototype was a 180-degree mechanical radial scanning instrument [23]. The first commercial EUS instrument, introduced in 1982, was a mechanical–radial type. The US transducer was located on the tip of the instrument and could be rotated by a motor within the endoscope handle. A 360-degree image was obtained perpendicular to the insertion shaft of the endoscope, which allowed for convenient, real-time mapping of the anatomy [24]. In the early 1990s, Pentax Medical, in cooperation with Hitachi, developed the first commercially available linear-array echoendoscope [23]. Linear echoendoscopes altered the landscape of EUS due to their ability to track a needle in real time, and across the image plane, into a targeted lesion. The electronic instruments also permitted the use of Doppler technology to assess vascular flow.

Around that time, the innovation of needle designs also began. Vilmann et al., in collaboration with Medi-Globe GmbH, created a special biopsy device, which was a major step leading to the clinical application of the biopsy method [25,26]. In 1992, these researchers reported the first case of using EUS-FNA to examine a pancreatic head lesion [25]. In 1993, they reported a further attempt, in which they used a newly developed steel needle with a Teflon sheath to examine upper-gastrointestinal tract lesions [27]. In 1996, Vilmann and Hancke reported the development of a new biopsy handle instrument (type Hancke/Vilmann) [28]. One year later, Binmoeller et al. first described an automated biopsy device for pancreatic lesions that could not be punctured with conventional aspiration needles [29]. The automatic spring-loaded biopsy needle allowed tissue sampling also of indurated pancreatic lesions. However, this instrument never attained success.

Thereafter, the range of indications for EUS-FNA expanded, and new clinical applications for biopsies other than conventional diagnostics were envisioned, including genetic diagnostics and anticancer drug-sensitivity assays. Such applications inevitably required the collection of an adequate tissue volume to achieve optimal diagnostic accuracy [30,31]. Therefore, the number of options for fine-needle biopsies (FNB) has increased in recent needle developments. FNB needles were especially designed to obtain core specimens with preserved tissue architectures. In 2002, Wiersema et al. reported initial experiences with EUS-FNB of perigastric organs [32].

**Figure 3:**
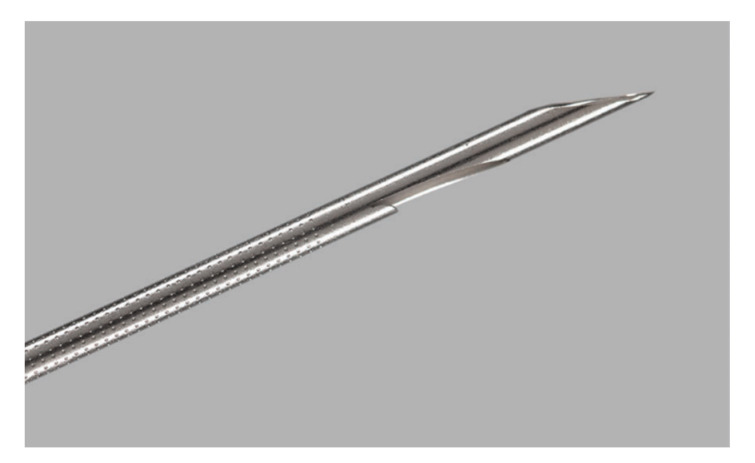
The 20G ProCore needle with an antegrade beveled side slot at the needle tip (Image courtesy of Cook Medical).

The initial core-biopsy EUS needle used was the 19-G Tru-Cut needle (Quick-Core; Cook Medical, Bloomington, IN, USA). Although in some cases, high diagnostic yields were obtained [33], the production of this needle was terminated due to its limited flexibility and occurring adverse events (AEs). The Tru-Cut was replaced by the ProCore FNB needles (Cook Medical) (Figure 3), which is characterized by a cutting bevel (reverse for 19-, 22- and 25-G, and 20-G antegrade beveled side slot) at the needle tip [34,35,36].

**Figure 4:**
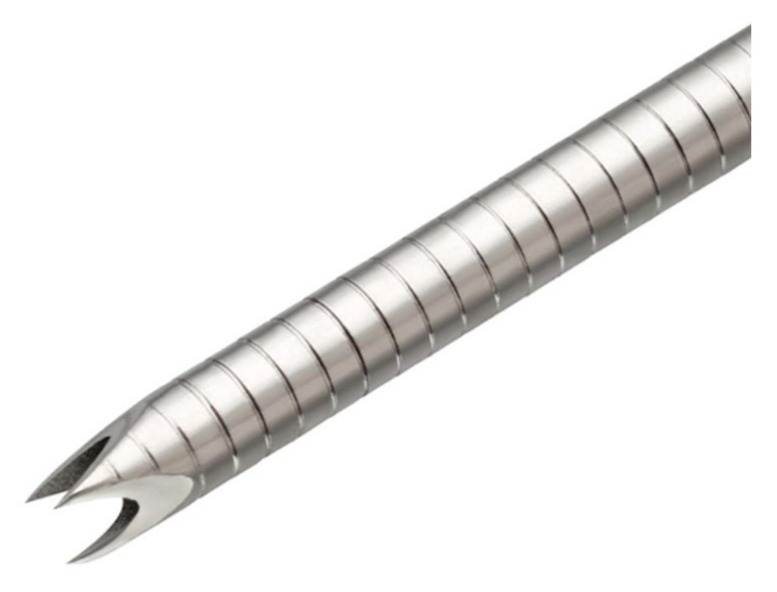
The franseen needle with three symmetric cutting tips (Image courtesy of Boston Scientific).

Recently, new FNB needles have been released in the market, including the fork-tip needle (SharkCore; Medtronic, Newton, Mass and Covidien, Dublin, Ireland), which is characterized by two sharp tips on the opposite side of the lumen [37], and the franseen-type needle (Acquire; Boston Scientific, Marlborough, MA, USA) (Figure 4), which is characterized by three symmetric cutting tips [38]. Currently, a variety of puncture needles that improve lesion accessibility and puncture performance are commercially available (Table 1).

## 3. Needle Size

At present, the most frequently used needle sizes are 22 G and 25 G. A 19-G needle ensures that a sufficiently large sample is collected for histologic diagnostics and immunostaining in cases that are examined for autoimmune pancreatitis and malignant lymphoma [10,39]. However, the greater puncture resistance encountered by these larger-sized needles increases the difficulty of the procedure. In particular, transduodenal puncture is thought to be difficult to perform [40]. Additionally, the larger the caliber of the needle, the higher the risk of post-puncture bleeding [41]. A smaller needle is technically more easy to handle within the device [42]. Therefore, 22-G or 25-G needles are more commonly used for EUS-FNA. Several studies have compared 22-G vs. 25-G needles (Table 2). In a review of a number of randomized clinical trials, one study showed a tendency towards higher accuracy of the 25-G needle [43], whereas the others showed a similar diagnostic yield of malignancy [44,45,46,47,48,49]. Studies comparing FNA with 22-G and 25-G needles were also reviewed as five meta-analyses [41,50,51,52,53]. However, more recent meta-analyses showed different results. Facciorusso et al. analyzed seven trials with 689 patients and 732 lesions (295 sampled with 22-G, 309 with 25-G, and 128 with needles of both sizes) [51]. A non-significant superiority of 25-G needles in pooled sensitivity (risk ratio: 0.93, 0.91–0.95, versus 0.89, 0.85–0.94 of 22-G needles; *p* = 0.13) and no difference in specificity (1.00, 0.98–1.00 in both groups; *p* = 0.85) were observed. Sample adequacy was similar between the two devices (risk ratio: 1.03, 0.99–1.06; *p* = 0.15). Very few AEs were observed and did not impact patient outcomes. This study showed no significant superiority of either group regarding pooled sensitivity, specificity, and safety.

On the other hand, Xu et al. analyzed eleven trials with 837 patients (412 sampled with 22- G vs. 425 with 25-G needles) for diagnosis of solid pancreatic lesions [52]. Their outcomes revealed that 25-G needles have a higher sensitivity than 22-G needles when used on solid pancreatic lesions EUS-FNA (92% (95% CI, 89–95%) versus 88% (95% CI, 84–91%); *p* = 0.046), although there were no significant differences between the two groups in overall diagnostic specificity (*p* = 0.842).

In 2018, Guedes et al. analyzed four randomized trials with 462 patients (229 sampled with 22-G vs. 233 with 25-G needles) for the diagnosis of solid pancreatic lesions. They found that the diagnostic sensitivity was 93% for 25-G and 91% for 22-G needles. In this study, the specificity of the 25-G needle was 87%, and that of the 22-G needle was 83%, with no statistically significant difference between the two needle types (*p* = 0.497). Based on randomized trials, this meta-analysis did not show a significant difference between the 22 G and 25 G needles [53]. As mentioned above, a range of opinions persist and no definitive recommendations can be made in favor of using one particular device or the other, as there appears to be no strong superiority of one needle over the other.

## 4. EUS-FNB

Several studies about diagnostic ability of FNB needles have been published. Early published results on the performance of ProCore FNB needles demonstrated high diagnostic accuracy [35,54,55]. Larghi et al. reported the first prospective, multicenter study in 61 patients in whom only a single needle pass with a 22-G FNB needle was performed [54]. The procedure was successfully performed in all but one patient, despite the fact that in 57% of cases, the puncture was carried out transduodenally. The rates of sensitivity, specificity, positive predictive value, negative predictive value, and accuracy for the histologic diagnosis of a pancreatic mass were 87.5, 100, 100, 41.7, and 88.5%, respectively. Two studies have compared the 20-G antegrade beveled biopsy needle with the 22-G and 25-G reverse beveled needles. According to these studies, the 20-G antegrade beveled biopsy needle is better than the reverse beveled needles when retrieving samples for histological analysis [56,57]. Moreover, a recent meta-analysis confirmed the superiority of the 20-G antegrade beveled biopsy needle compared with reverse beveled needles [58].

A large and early retrospective multicenter study of EUS-FNB obtained by fork-tip FNB needle on different solid lesions (pancreatic, SEL, and lymph-node) demonstrated an excellent 88% overall pathologic diagnostic yield based on a median of only two passes [59]. Overall, histological diagnostic ability and thus pathologic yield for the different lesion subtypes were as follows: pancreatic lesions 86%, SELs 87%, lymph nodes 93%. Needle size did not affect pathologic diagnostic yield, with both 25-G and 22-G needles delivering high yields of 86% and 89%, respectively. The 25-G fork-tip FNB needle demonstrated high diagnostic accuracy (93%) in a recent multicenter prospective study [60].

A retrospective multicenter study of 200 patients undergoing EUS-FNB of solid lesions (pancreas, SEL, lymph node, and other) using franseen needles also showed a high rate of tissue adequacy and tissue core [61]. The tissue obtained by these needles was adequate for evaluation and diagnosis by ROSE in 98.5% of cases. In 131/145 (90%) of cases, a core of tissue was obtained.

Further studies comparing outcomes based on fork-tip versus franseen needles could determine which needle design is superior in terms of diagnostic yield and safety. For pancreatic lesions, one comparative was reported [62]. Sampling was performed twice in 50 patients using the two needle types in randomized order. The results showed comparable diagnostic yields (96% vs. 92%, *p* = 0.32) and diagnostic adequacy with ROSE (94% vs. 96%, *p* = 0.32) of the franseen and fork-tip needles, respectively. In addition, no statistically significant differences were found in terms of tissue quality or quantity, total tissue obtained, total tumor tissue, and the desmoplastic fibrosis yield obtained by the two needles. Another randomized controlled trial also demonstrated no significant difference in the diagnostic accuracy, 64 out of 75 (85.3%) versus 68 out of 75 (90.7%), (*p* = 0.45) between franseen and fork-tip needles [63]. Recently, a meta-analysis comparing the two needles for EUS FNB of solid mass lesions was published [64]. The analysis featured a total of 21 studies with 1632 patients. The pooled diagnostic yield with fork-tip needle was 92.8% (95% CI 85.3–96.6, *I*2 = 73.1), whereas the pooled diagnostic yield using the franseen needle was 92.7% (95% CI 86.4–96.2, *I*2 = 88.4), demonstrating no statistical difference between the needles (*p* = 0.98). The overall rate of AEs for both needles combined was 4.2% (95% CI 2.8–6.4, *I*2 = 0.0); for the fork-tip and franseen needles, the rates were 3.7% (95% CI 2.3–6.0, *I*2 = 0), and 6.2% (95% CI 2.6–14.1, *I*2 = 0.0), respectively (*p* = 0.31, not significant). Of note, the AE rates of EUS-FNB were slightly higher than what would be anticipated, given that EUS-FNA is generally considered to be very safe. It is theoretically possible that the novel design of these FNB needle tips may allow for more effective capture of the tissue prior to shearing it off, but that this may induce more localized trauma, resulting in more frequent AEs.

Thus far, no consensus has been reached regarding optimally effective and safe needle types. Therefore, a need exists to conduct larger, multicenter, randomized trials to evaluate subtle advantages and disadvantages among new FNB needles.

ROSE on EUS-FNB is performed using the touch imprint cytology. The acquired material from the EUS-FNB was placed on a smear and examined for the presence of a solid component. The worm-like core tissue was then separated from the bloody material and was transferred to a clean slide and gently pushed and rubbed down with another slide. The remaining solid tissue from the first pass (if available) was placed in formalin for histological evaluation [65]. A large international randomized trial investigating the usefulness of ROSE coupled with EUS-FNB vs. EUS-FNB alone is ongoing [66].

## 5. EUS-FNB vs. EUS-FNA

Several randomized controlled trials have compared diagnostic yields of FNA and FNB. Alatawi et al. performed a randomized controlled trial to compare the outcomes of the 22 G Procore FNB-versus the 22-G FNA needle in patients with solid pancreatic lesions [67]. They included 100 patients who were equally allocated to FNA and FNB groups. Sampling outcomes showed a significantly lower adequate sample rate in the FNA than in the FNB group (90% vs. 100%, *p* = 0.02). Additionally, this study showed that the use of Procore FNB needles can attain a high diagnostic performance after two needle passes, and that additional passes do not significantly improve the diagnostic yield. FNA needles may achieve the same efficacy for the cytological diagnosis of pancreatic cancer, but this requires a higher number of needle passes. The researchers concluded that the samples obtained with 22-G Procore FNB needles were of significantly higher histological quality and required fewer needle passes for diagnosis. Another randomized controlled trial was conducted by Kim et al. [68]. Their study featured a comparison of the 22-G Procore FNB- and the 22-G FNA needles in patients with SELs. Compared to the EUS-FNA group, the EUS-FNB group required a significantly lower number of needle passes to obtain macroscopically optimal core samples (median: 4 vs. 2, *p* = 0.025). Moreover, compared to EUS-FNA needles, EUS-FNB needles produced higher yields of macroscopically and histologically optimal core samples after three needle passes (30% vs. 92%, *p* = 0.006, and 20% vs. 75%, *p* = 0.010 respectively). Finally, a higher diagnostic accuracy was obtained with EUS-FNB than with EUS-FNA needles (75% vs. 20%, respectively; *p* = 0.010). The authors concluded that EUS-FNB has a superior ability to obtain histological core samples and also a higher diagnostic accuracy than EUS-FNA for SELs.

A recent systematic review and meta-analysis compared the diagnostic yields of FNA and FNB in patients with lesions of the pancreas, lymph nodes, or solid GI lesions, while specifically evaluating the diagnostic value of ROSE [69]. Fifteen studies (*n* = 1024) were included in the analysis. No significant difference was observed in diagnostic adequacy (defined as the ability to procure cytological and/or histological samples adequate for interpretation) (Relative risk (RR): 0.98, CI: 0.91-1.06, *I*2 = 51%). In the absence of ROSE, FNB showed a trend toward better diagnostic adequacy, which, however, fell short of statistical significance (*p* = 0.06). Only in patients with solid pancreatic lesions, no difference in diagnostic adequacy was observed (RR: 0.96, CI: 0.86–1.09, *I*2 = 66%), but, in the absence of ROSE, FNB was associated with a higher diagnostic adequacy (*p* = 0.02). No significant differences were found in diagnostic accuracy (RR: 0.99, CI: 0.95–1.03, *I*2 = 27%) and procurement of optimal-quality core histological samples (RR: 0.97, CI: 0.89–1.05, *I*2 = 9.6%). However, FNB enabled the diagnosis with fewer passes (Standardized mean: 0.93, CI: 0.45–1.42, *I*2 = 84%) (Table 3).

Given the above results, FNB without ROSE could replace FNA with ROSE without loss of diagnostic accuracy. So, in medical centers where ROSE has not been applicable, FNB may still be an effective option.

## 6. Interventional EUS

EUS is clinically useful not only as a diagnostic tool but also during interventions. Many interventional EUS procedures have replaced conventional therapeutic approaches in the treatment of various gastrointestinal diseases. In fact, this procedure has emerged as the treatment of choice for management of pancreatic fluid collections (PFC) and drainage of mediastinal and intra-abdominal abscesses. Similarly, EUS-guided biliary drainage (EUS-BD) is applied in patients with failed cannulations and inaccessible papillae, as well as in those with duodenal/gastric obstructions or surgically altered anatomies. In addition, EUS-guided gall bladder drainage may gain prominence as a useful procedure in patients with acute cholecystitis who are unfit for surgery [70]. While interventional EUS has clinical benefits in these patients and demonstrates high technical and clinical success rates in high-volume centers, its use is associated with various AEs, including stent migration. If stent migration does occur, it is occasionally fatal [71]. In this sense, interventional EUS can be a high-risk procedure, and only endoscopists skilled in both ERCP and EUS should be permitted to perform it. Clearly, there is a practical need for the establishment of techniques and devices that are more feasible and safer.

**Figure 5:**
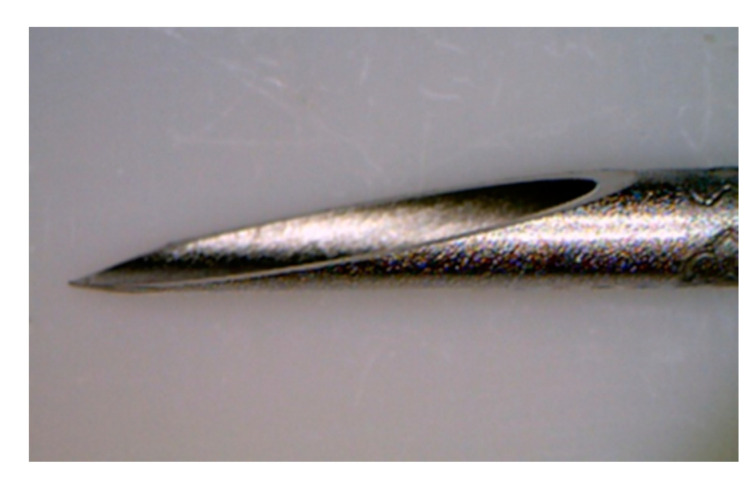
The 19 G Sono Tip Pro Control (Medi-Globe GmbH) with a cut-surface length of 5 mm.

A 19 G or 22 G FNA needle or needle knife is used to puncture the target when interventional EUS is performed. To enable passage of 0.035- or 0.025-inch guidewires through a needle, a 19-gauge needle should be selected. The SonoTip Pro Control 19 G needle (Medi-Globe GmbH, Rosenheim, Germany) has been reported as a suitable needle to perform interventional EUS (Figure 5). Since the needle is highly flexible, it is easy to adjust the insertion angle of the needle. In addition, the cutting surface of this FNA needle is 5 mm long and is considered to be extremely sharp, so it is easy to operate the guidewire without kinking. Therefore, the needle is considered to be appropriate for use in interventional EUS [72]. However, when attempting to puncture an intrahepatic bile duct at a distant position, the highly flexible needle may not move straight and may deviate from its target. In such cases, it may be necessary to select another needle.

**Figure 6:**
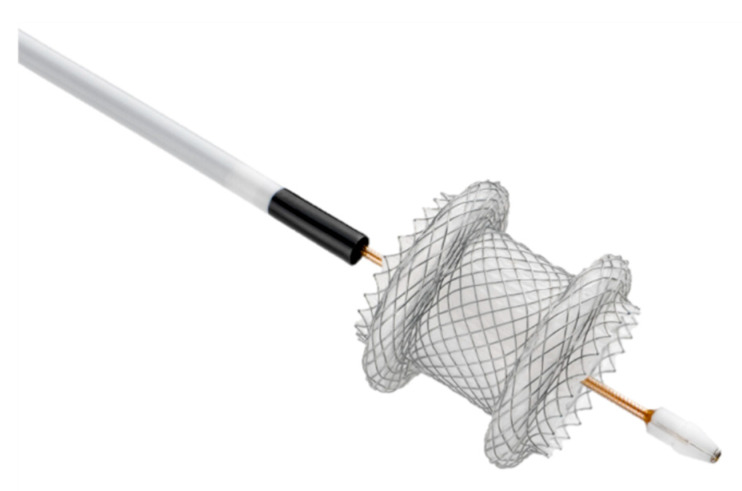
Hot-Axios stent delivery system with electrocautery at the distal tip of the delivery catheter (Image courtesy of Boston Scientific).

Interventional EUS continues to evolve as new devices dedicated to this technique are being developed. One of the greatest recent impacts in this field has been the emergence of a new stent delivery system integrated with an electrocautery needle. In 2014, Teoh et al. reported the testing of a new device dedicated to interventional EUS, which creates a fistula by electrocautery followed by placement of a fully covered metal stent in one procedure (Hot-Axios; Boston Scientific, Marlborough, MA, USA) (Figure 6) [73]. This device is a solution for endoscopists to tackle different complex tasks, i.e., to safely dilate the puncture tract, and to steadily and precisely deploy a stent in many EUS-guided interventions. The device has also prompted further refinement of procedures and the introduction of new techniques [74]. Since its introduction into the market, many reports on interventional EUS have been published based on this device. Its most common application is drainage for PFC, including the treatment of walled-off necrosis. Walter et al. reported the results of a prospective cohort study of 61 cases with PFC in which this lumen-apposing metal stent was used. The results of this study confirmed the stent’s feasibility and good clinical outcomes with low AE rates [75]. Several retrospective studies with larger numbers of cases have already shown favorable technical and clinical outcomes. Nevertheless, further evaluation is still needed of the relative efficacy and the cost–benefit ratio of this technique through comparisons with established techniques such as double pig-tail stent deployment. Future improvements might include the development of a dedicated device for EUS-BD, similar to those such as Hot-Axios that is optimized to achieve puncture and stent placement in one step, to prevent migration.

## 7. The Use of Through-the-Needle Imaging

Confocal laser endomicroscopy (CLE) is a novel imaging method that allows microscopy of the gastrointestinal mucosal epithelium during endoscopy, enabling real-time optical biopsies [76]. Needle-based confocal laser endomicroscopy (nCLE) is a promising endoscopic diagnostic technique that can be delivered through a 19-G needle into lesions and provides real-time, in vivo, 500–1000x magnified microscopic tissue imaging during EUS [77]. Fluorescein is usually administered immediately before imaging, and optimum images are obtained between 30 s to 8 min after injection but can be interpreted for as long as 60 min [78]. Recent studies have revealed the diagnostic value of EUS-guided nCLE for various lesions.

According to several studies, a classification of nCLE patterns could be established, facilitating the diagnosis of pancreatic cystic lesions [79]. A recent prospective multicenter study of 206 patients with pancreatic cystic lesions showed thay sensitivities and specificities of nCLE for the diagnosis of serous cystadenomas, mucinous cystic neoplasms, and premalignant cystic lesions were all 0.95 or higher; the area under the receiver operating characteristic curve was significantly larger for nCLE than for cyst fluid carcinoembryonic antigen or EUS [80]. A number of studies also evaluated the feasibility and safety of nCLE for the assessment of solid organs (i.e., solid pancreatic lesions, lymph nodes, and SELs) [81,82,83]. Although in vivo real-time imaging using nCLE is highly concordant with pathology and may replace ROSE, it is important to keep in mind that EUS-FNA alone has a sensitivity around 90% for malignancy. Therefore, it is questionable whether the adjunct of nCLE can improve the diagnostic value for solid mass any further.

Although nCLE may be used routinely for diagnosing pancreatic cysts in the future, there are several limitations to this technology. During real-time nCLE diagnosis, blinding the endoscopist to EUS features of the tumor was impossible, which inevitably affected the diagnosis of real-time nCLE [77]. Additionally, heterogenicity in endoscopist’s experience, inter-observer variability, and reproducibility of nCLE image should be taken into account [84]. To justify routine use of nCLE, advanced training and validation are needed.

Another through-the-needle imaging technique is EUS-guided through-the-needle biopsy (EUS–TTNB). The instrument has a sheath diameter of 0.8 mm and a jaw-opening width of 4.3 mm. It can be introduced through a 19-G FNA needle, after puncturing a pancreatic cystic lesion. Consequently, it enables the collection of histological specimens from the pancreatic cyst wall. Interobserver agreement between expert pathologists in the evaluation of TTNB samples from pancreatic cystic lesions has demonstrated by Larghi et al. [85]. It was close to perfection for all evaluated parameters, except definitive diagnosis. When mucinous cystic lesions were compared versus all other diagnoses, the agreement became substantial, thus indicating that TTNB specimens can provide important information for pancreatic cystic lesions. Recently, a multicenter retrospective study of this intervention was published, which featured 28 patients. The technical and clinical success rates of this technique were found to be 85.7% and 71.4%, respectively. Technical failure seems to be caused by loss of flexibility of the echoendoscope when forceps are inserted. Even though no severe or fatal AEs were observed, a rate of 10.7% is notable and should be interpreted with caution because of the limited number of patients. One patient was hospitalized due to nonspecific abdominal pain and was discharged the following day after the symptoms resolved. Two patients suffered acute pancreatitis in mild forms and hospitalized for 4 and 6 days [86]. Larger retrospective studies have demonstrated a high diagnostic yield. Crinò et al. evaluated 61 patients, and the diagnostic reliability of TTNB sampling compared with surgical histology was 90% [87]. Barresi et al. evaluated 56 patients, and overall diagnostic yield by combining cytological and histological samples was 47/56 (83.9%, 95% CI, 72–92%) [88]. Yang et al. performed a prospective study of 114 patients and compared EUS-FNA with TTNB. In their study, samples collected by EUS-FNA were analyzed by cytology, and samples collected by TTNB were analyzed by histology. Tissue acquisition yield was significantly higher with TTNB (95 of 114, 83.3%) than FNA (43 of 114, 37.7%) *(p* < 0.001). Findings from samples collected by TTNB were 100% concordant with findings from surgical specimens (14 of 14), whereas only 3 of 14 findings from analysis of samples collected by FNA were in agreement with findings from surgical specimens (21.4%) (*p* < 0.001) [89]. Moreover, three meta-analyses comparing EUS-FNA with TTNB were published. The pooled diagnostic accuracy rate, sensitivity, and specificity of TTNB were 78.8%, 82.2%, and 96.8%, respectively. The AEs rates were 6.1–8.6% [90,91,92].

The possibility of obtaining histological specimens from the cyst wall when using this novel microbiopsy forceps could lead to drastic changes in the management algorithm of pancreatic cysts if it can be performed safely. If biopsy specimens extracted through this procedure contain sufficient material for supplementary immunohistochemical (IHC) analysis, this will enable specialists to reach more accurate histological diagnoses. In cases of intraductal papillary mucinous neoplasm (IPMN), histological subtyping will become a possibility. When compared to gastric and oncocytic subtypes, pancreatobiliary and intestinal subtypes are associated with progression to high-grade dysplasia and invasive carcinoma [93]. Previous surgical series have shown that the clinical behavior of invasive carcinomas derived from pancreatobiliary type IPMN has a significantly poorer prognosis than carcinomas of the intestinal subtype [94,95]. In addition, procurement of cellular material from the cysts enables next-generation sequencing (NGS) and testing for possible gene mutations. When combined with microbiopsy and IHC, NGS could potentially increase diagnostic accuracy and lead to a personalized approach when deciding on a treatment strategy. Prospective multicenter studies are needed to determine the feasibility of its more widespread use in clinical practice.

## 8. Conclusions

This article has summarized the historical advances in the design and performance of needles used for EUS-guided procedures. Since the early 1990s, EUS-FNA has played a clear and vital role in oncological care and has become the standard procedure for sampling tissues with high diagnostic accuracy. Although there are diverse opinions and no definitive recommendations regarding the preferred use of one particular device, FNB may provide further information on tissue architecture and provides a greater sample yield, which allows for further analyses, such as genetic sequencing and phenotyping. This again may enable more personalized treatment strategies.

Moreover, other EUS-guided procedures such as interventional EUS and through-the-needle devices have been developed. As mentioned above, there are continuing attempts to improve the diagnostic ability and therapeutic technique, which will benefit from a better understanding and the selection of the most appropriate types of puncture needles.

## Figures and Tables

**Table 1:** List of needles used for endoscopic ultrasound-guided procedures.

Release Year	Needle	Size	Material of Needle
2000	Echo Tip Ultra (Cook Medical)	19, 22, 25	Stainless
2001	NA-11J-KB (Olympus Medical Systems)	22	Stainless
2003	EZ Shot (Olympus Medical Systems)	22	Stainless
2004	Quick-Core (Cook Medical)	19	Stainless
2011	Expect (Boston Scientific)	19, 22, 25	Cobalt-chromium
2011	EZ Shot 2(Olympus Medical Systems)	19, 22, 25	Stainless
2012	Echo Tip Procore (Cook Medical)	19, 22, 25	Stainless
2012	SONO tip Pro Control (Medi-Globe GmbH)	19, 22, 25	Stainless
2012	Expect 19 G Flex Needle (Boston Scientific)	19	Nitinol
2013	EUS Sonopsy CY (HAKKO)	21	Stainless
2016	EZ Shot 3 Plus (Olympus Medical Systems)	19, 22	Nitinol
2016	Echo Tip Procore 20 G (Cook Medical)	20	Stainless
2016	SharkCore (Medtronic)	19, 22, 25	Stainless
2016	Acquire (Boston)	22, 25	Cobalt-chromium
2017	Acquire 19 G Flex Needle (Boston Scientific)	19	Nitinol
2018	EZ Shot 3 Plus (Olympus Medical Systems)	25	Stainless
2020	SONO tip TopGain (Medi-Globe GmbH)	19, 22, 25	Stainless

**Table 2:** Published comparative studies regarding 22 G versus 25 G FNA-needles.

Reference	Study Design	Cases (n)	Lesion	Rose	Diagnostic Ability (25G vs. 22G)
Lee [44] 2009	RCT	12	Mainly pancreas	Yes	n.s
Siddiqui [45] 2009	RCT	131	Pancreas	Yes	Accuracy; 95.5% vs. 87.5%; *p* = 0.18; n.s
Camellini [46] 2011	RCT	127	Mainly pancreas	Yes	Accuracy; 78.1% vs. 77.8%; n.s
Fabbri [47] 2011	RCT	50	Pancreas	Yes	Accuracy; 94% vs. 86%; n.s
Lee [48] 2013	RCT	188	Pancreas	No	Accuracy; 88.3% vs. 89.4%; *p* = 0.82; n.s
Vilmann [49] 2013	RCT	135	Pancreas and LN	No	Sensitivity; 94.1% vs. 94.1%; n.s
Carrara [43] 2016	RCT	144	Mainly pancreas	Yes	Accuracy; 81% vs. 68%; *p* = 0.09; n.s
Affolter [41] 2013	MA (11 studies)	1452	Mainly pancreas	5/11	Sensitivity; 91% vs. 78%; *p* = 0.97; n.s
Madhoun [50] 2013	MA (8 studies)	1292	Pancreas	5/8	Sensitivity; 93% vs. 85%; *p* = 0.0003
Facciorusso [51] 2017	MA (7 studies)	732	Pancreas	5/7	Sensitivity; 93% vs. 89%; *p* = 0.13; n.s
Xu [52] 2017	MA (11 studies)	837	Pancreas	6/11	Sensitivity; 92% vs. 88%; *p* = 0.046
Guedes [53] 2018	MA (4 studies)	462	Pancreas	2/4	Sensitivity; 93% vs. 91%; *p* = 0.497; n.s

RCT, randomized controlled trial; MA, meta-analysis; n.s, not significant.

**Table 3:** Published comparative studies regarding EUS-FNA versus EUS-FNB.

Reference	Study Design	Cases (n)	Lesion	Diagnostic Ability (FNA vs. FNB)
Alatawi [67] 2015	RCT	100	Pancreas	Adequate sample rate; 90% vs. 100%; *p* = 0.0003
Kim [68] 2014	RCT	22	SELs	Accuracy; 20% vs. 75%; *p* = 0.010
Khan [69] 2017	MA (15 studies)	1024	pancreas, LN and solid GI lesion	FNB showed better diagnostic adequacy without ROSE; *p* = 0.02

RCT, randomized controlled trial; MA, meta-analysis.
